# The Knee-SCHOOL: a brief patient-centered multidisciplinary educational program for knee osteoarthritis

**DOI:** 10.3389/fmed.2024.1497774

**Published:** 2025-01-03

**Authors:** Denise Vianna Machado Ayres, Sabrina Saemy Tome Uchiyama, Andréa Oliveira Prates, Rosana Aparecida Freitas Lopes, Antenor Bispo Santos Silva, Denise Rodrigues Tsukimoto, Rosimeire Alves Amorim, Taynah Souza Ribeiro, Artur Cesar Aquino Santos, André Tadeu Sugawara, Marcos Montagnini, Linamara Rizzo Battistella, Marta Imamura

**Affiliations:** ^1^Instituto de Medicina Fisica e Reabilitacao, IMREA, Hospital das Clínicas HCFMUSP, Faculdade de Medicina FMUSP, Universidade de São Paulo, São Paulo, Brazil; ^2^Departamento de Medicina Legal, Bioetica, Medicina do Trabalho e Medicina Fisica e Reabilitacao, Faculdade de Medicina da Universidade de São Paulo, FMUSP, São Paulo, Brazil; ^3^Division of Geriatric and Palliative Medicine, University of Michigan, Ann Arbor, MI, United States

**Keywords:** knee osteoarthritis, education, rehabilitation, pain, self-management

## Abstract

**Background:**

Knee osteoarthritis (KOA) is the most common form of arthritis in adults and a leading cause of years lived with disability, representing a significant burden on healthcare worldwide.

**Objective:**

Describe the structure and educational elements of the Knee-SCHOOL, a brief patient-centered multidisciplinary educational program for patients with KOA.

**Design:**

Observational prospective study.

**Setting:**

Academically affiliated rehabilitation outpatient center in Brazil.

**Methods:**

The program consisted of three in-person educational sessions (4.5 hr each) for 55 community dwelling adults, aged ≥50 years, with primary KOA-related pain. Study measures included demographic data (age, sex, and educational level), pain duration (years), pain intensity (visual analogue scale), affected knee (right, left, or both knees), comorbidities (presence of hypertension, diabetes, and hypercholesterolemia), Body Mass Index (BMI), Bristol Stool Scale, Adapted Healthy Eating Index (AHEI), bioelectrical impedance, daytime sleepiness, and the impact of the KOA on pain, symptoms, activities of daily living, recreation, and quality of life. Participants attended educational sessions delivered by a multidisciplinary team (two physicians, two nurses, two physical therapists, one occupational therapist, one dietitian, one psychologist, one social worker, and one physical educator) addressing several aspects of KOA. They also participated in supervised exercise practice and a home exercise program.

**Results:**

Fifty-five subjects completed the study. The mean age was 67.73 (± 7.73) years; most were females (70.9%), 92.7% had bilateral KOA, with mean pain duration of 12.41 (± 10.17) years. The mean BMI was 32.52 (± 5.99), 65.5% were obese, and 96.4% reported an inadequate diet. KOA had a more negative impact on sports, recreation and quality of life. Daytime sleepiness was uncommon. The mean pain intensity, measured with visual analogue scale, score reduced from 5.52 (± 2.11) at baseline to 4.04 (± 2.38) after the program (week 2). The effect size was 0.7 (95% CI 0.32 to 1.07). All participants received the program well, with no drop-out rates or reported adverse events.

**Conclusion:**

The Knee-SCHOOL utilized a multidisciplinary educational approach and an exercise practice addressing multiple aspects of KOA pain. While more studies are needed to assess the longitudinal impact of the program, it was promising in managing pain.

## Introduction

Knee osteoarthritis (KOA) is the most common type of arthritis in adults and a leading cause of years lived with disability (1). It also represents a significant financial burden on healthcare systems worldwide (1). The global prevalence of KOA was approximately 600 million in 2020 and is estimated to increase by almost 75% in 2050 (1). In addition to chronic pain and physical disability, patients with KOA frequently experience sleep problems (2, 3) and psychological symptoms, including depression and anxiety (4, 5). Population studies demonstrate a higher prevalence of KOA in women, older patients, and patients with obesity (6–8). In addition, there are increased cardiovascular risk factors in patients with KOA (9–12).

The overall management of KOA includes proper analgesia and improved function and quality of life. Pharmacological interventions for KOA-related pain include topical analgesics (such as diclofenac gel and capsaicin), oral analgesics (like non-steroidal anti-inflammatory drugs (NSAIDs), acetaminophen, paracetamol, and duloxetine), and intra-articular therapy (such as steroids and hyaluronic acid) (13). Non-pharmacological agents including complementary and integrative health interventions such as acupuncture (14), meditation and yoga (15), tai chi (16), and transcutaneous electrical nerve stimulation (17) improve pain and function in patients with KOA. Self-management is considered a mainstay approach for the management of osteoarthritis (OA) (18). It involves the patient actively managing the chronic condition. Self-management can be achieved through educational interventions focused on enhancing the understanding of the disease, pain management, establishing an exercise routine and activity pacing, weight loss, sleep, and other modifiable risk factors, including joint alignment and joint preservation (19–22).

Several studies demonstrated the benefits of patient education and exercise therapy in osteoarthritis-related pain (23–25). Hansson et al. (26) demonstrated that patient education is feasible and can improve self-perceived health and function in primary care patients with KOA. In addition, recent systematic reviews documented that patient education effectively reduces pain related to knee and hip OA (23), and combination therapy of exercise and education interventions improves physical activity and pain reduction in knee and hip OA (24, 25).

KOA represents a significant health system burden in Latin America (27). In Brazil, KOA has become a significant public health concern due to the growth of the aging population. It is estimated that 9.6% of Brazilians over 39 years of age suffer from KOA (28). Despite being one of the largest public health systems in the world (29), Brazil has historically lacked public health policies addressing the management of OA in the general population (30). In 2018, the Brazilian Ministry of Health launched a health systems research initiative focusing on interventions to improve the care of individuals with disabilities. Researchers from our institution, the Instituto de Medicina Física e Reabilitação (Instituto de Medicina Física e Reabilitação do Hospital das Clínicas da, Faculdade de Medicina da Universidade de São Paulo - IMREA HCFMUSP), successfully obtained grant funding under this governmental initiative to develop a clinical trial for studying interventions for KOA-related pain. The initial phase of this clinical trial involved implementing a multidisciplinary educational program for managing pain in a sample of community-dwelling adults with chronic pain related to KOA, the Knee- SCHOOL.

The goal of this paper is to describe the structure and educational elements of the Knee-SCHOOL. We also explore the impact of this program on reducing pain in patients with KOA.

## Materials and methods

This is a descriptive and observational prospective study of a multidisciplinary educational program for community-dwelling adults with KOA. The study is part of a major Randomized Clinical Trial (RCT) conducted at IMREA HCFMUSP, a teaching hospital of the University of São Paulo School of Medicine and approved by the Institution Review Board (CAAE: 0875619.8.000.0068). IMREA is accredited by the Commission on Accreditation of Rehabilitation Facilities (CARF) and fully integrated into the public health system, providing tertiary care in an academic setting.

Study participants were recruited from the institution’s patient registry and the community. In addition, patients referred to IMREA for pain related to KOA were screened to participate in the study. Study investigators assessed eligibility for the study by reviewing medical records, radiologic data, and by patient interviews.

The inclusion criteria included patients older than 50 years and with moderate to severe pain related to KOA for at least 3 months duration who met the American College of Rheumatology (31) and the Kellgren-Lawrence radiographic grading criteria (32) for primary KOA.

The exclusion criteria included patients with severe psychological or psychiatric diseases, fibromyalgia, systemic inflammatory rheumatic diseases and neoplasia. Patients with other forms of KOA (e.g., post-traumatic, inflammatory and hemosiderosis arthritis) were excluded from the study. Participation was voluntary and all patients provided informed consent before enrollment.

Study measures included demographic (age, sex, and educational level), pain duration and intensity, affected knee (right, left, or both knees), comorbidities, and Body Mass Index and other nutritional measures, daytime sleepiness, and the patient’s rating of the KOA on five domains: pain, symptoms, activities of daily living, sports/recreation activities, and quality of life. Frequency of attendance was captured and recorded as patient participation.

Pain intensity was assessed using a 10-cm visual analogue scale (VAS) at the beginning and end of the program. The participants were instructed to mark their perceived pain intensity on the scale (zero: no pain, 10: the worst possible pain).

The Knee injury and osteoarthritis outcome score (KOOS) (33) was used to evaluate patient’s opinion about their knee and associated problems. It is a self-administered questionnaire that evaluates five outcomes related to KOA: pain, symptoms, and activities of daily living, sports /recreational activities, and quality of life. The scoring is based on a 0 to 100 scale, where zero represents severe knee problems and 100 indicates no knee issues.

The nutritional measures included information on Body Mass Index (BMI), bowel habits based on the Bristol scale (34), Adapted Healthy Eating Index (AHEI), bioelectrical impedance, and comorbidities (diabetes, hypertension, and hyperlipidemia).

The nutritional status was classified based on the BMI cut-off points adopted by the Pan American Health Organization (PAHO) for the SABE Study: ≤23 kg/m^2^ = underweight; >23.0 and < 28.0 kg/m^2^ = normal weight; ≥28.0 and < 30.0 kg/m^2^ = overweight; ≥30.0 kg/m^2^ = obesity (35).

The Bristol Stool Scale is a tool used to assess the shape and consistency of stool; the scale categorizes stool into seven types based on shape and consistency (34). Stool shape and consistency is categorized as: Type 1: Separate small lumps, Type 2: Sausage-shaped with irregular surface. Type 3: Sausage-shaped with smooth surface. Type 4: Smooth sausage or cylinder. Type 5: Ball or oval-shaped. Type 6: Flaky or fragmented. Type 7: Liquid or completely unformed. Types 3 and 4 are considered normal.

The Avanturi nutritional analysis software program was used to calculate the 24-hour food record (Santana RI. Avanturi: nutritional assessment software, version 4.0. Rio de Janeiro, 2009). Eating habits were calculated using the adapted Brazilian version of the Healthy Eating Index (36). This index evaluates 12 components, including food groups such as cereals, fruits, vegetables, legumes, meats, dairy products, oils and fats, sweets and sugars, as well as nutrients (total fat, saturated fat, and cholesterol) and food variety. Diets scoring below 71 points on the AHEI are classified as poor quality, while those between 71 and 100 need improvement, and above 100 are considered good quality (36). Poor quality and need improvement categories were considered as inadequate diets.

A bioelectrical impedance test was also performed using the InBody 370S device (Ottoboni, Rio de Janeiro, Brazil). BMI, fat percentage, and skeletal muscle mass index (SMI) were calculated as body composition measures.

The Epworth Sleepiness Scale (ESS) (37) was used to evaluate the degree of daytime sleepiness. It is a self-administered questionnaire that assesses the probability of falling asleep in eight everyday situations. The scoring for each item varies from 0 (no chance of napping) to 3 (great probability of napping). The total score is based on a scale of 0 to 24. A score of ≥11 was defined to indicate daytime sleepiness.

### Program structure

The Knee-SCHOOL consisted of three face-to-face educational sessions of 4.5 hr each over two consecutive weeks. Each session was limited to ten participants. The sessions included presentations by the members of the multidisciplinary research team, including two Physical Medicine and Rehabilitation physicians, two nursing professionals, two dietitians, two physiotherapists, one occupational therapist, one psychologist, one social worker, and one physical educator. The sessions also included supervised exercise practice; all participants received a prescription for home exercises. Table 1 describes the educational content of each session.

**Table 1:** Educational content of the Knee-SCHOOL.

Team member	Session 1	Session 2	Session 3
Physician	Participants received printed material (booklet) (https://redelucymontoro.org.br/site/area-do-paciente/materiais-educativos/acesse-aqui-os-videos-da-cartilha-cuidando-do-seu-joelho/) containing relevant information from all disciplines. Review of the program’s goals and objectives. Discussion of patients’ expectations. Definition of KOA and progression; modifiable and non-modifiable risk factors; symptomatology; anatomy of the knee; treatment options and benefits; pathophysiology of chronic pain; hypersensitivity of the nervous system; and the impact of pain in function. Duration: 60 min.		
Physiotherapist	Education on stretching exercises of the posterior muscle groups of the lower extremities, isometric exercises for the hip abductors and adductors, and isometric exercises for the knee extensors; three daily sets of ten repetitions. Discussion of exercise techniques based on pain’s intensity and the patient’s experience with physical activity. Duration: 30 min	Exercise practice (direct instruction using an elastic band for stretching and a plastic ball for isometric exercises; booklet review). Duration: 30 min	Same exercise practice as Session 2. Instruction on independent exercise practice at home and in the community using digital resources available on IMREA’s website. https://redelucymontoro.org.br/site/area-do-paciente/materiais-educativos/acesse-aqui-os-videos-da-cartilha-cuidando-do-seu-joelho/ Duration: 30 min
Occupational Therapist	Discussion of the impact of KOA on pain, quality of life, healthy aging, activities of daily living, mobility, and work-related activities. Duration: 30 min	Instruction on adaptation strategies for daily activities. Education about posture and positioning during occupational performance, environmental modifications, and the use of assistive technology for maintenance and promotion of function. Duration: 60 min	
Nursing Professional	Education on health promotion and sleep hygiene. Sleep hygiene consisted of education on establishing a regular sleep routine, such as going to bed and waking up at the same time, creating an optimal sleep environment (dark, quiet room with comfortable temperature), and avoiding light stimuli, such as electronic device screens, before bedtime, regulating diet and fluid consumption, especially stimulants, and regular physical activity. Patient education and skill development on self-care. Duration: 30 min	Recognizing limitations in essential physiological functions, including bladder function and sleep quality. Establishing life goals based on constraints. Patient education on pain, sleep, and sleep hygiene practices. Duration: 30 min	
Dietitian	Education on a healthy diet based on typical foods available in the patient’s environment and seasons. Discussion of the food pyramid using an educational tool. Education on food preparation, portions, categories, and label reading. Duration: 30 min	Review of the food education tool. Education on food portions, preparation, processed foods, and healthy food choices. Duration: 30 min	Education on establishing a healthy diet and food choices based on the presence of comorbidities (e.g., diabetes, hypertension, dyslipidemia, etc.). Patients with diabetes and dyslipidaemia were advised to choose whole grains, avoid sugars, replace them with natural sweeteners, and avoid low-fiber carbohydrates. Patients were also instructed to select adequate vegetable intake, drink water instead of sweetened beverages, low-fat dairy products, and lean meats, and divide meals throughout the day, avoiding long periods of fasting. In addition to the above guidelines, patients with hypertension were encouraged to reduce salt intake to no more than five grams/day. Avoid pre-packed seasonings and replace them with natural choices. Guidance on using healthy seasonings, recipes, and tasting of herbal salt. Education about lean meats, dairy products, and reading food labels. Duration: 60 min
Psychologist	During psychoeducational care, patients were instructed on the characteristics, personal meaning, and emotional aspects involved in the pain mechanism (anxiety, fear, irritability, and depression) and how to deal with negative emotions. Duration: 30 min	In the second session, patients were led to reflect on the beliefs that negatively influence pain treatment and their sense of control. They were also encouraged to exercise self-empowerment as a pain management and quality of life skill, and to review of the impact of their own belief system on their life experiences. Duration: 30 min	Relaxation practice to promote greater body awareness. Reflection on the experience of being part of the group. Discuss expectations, adherence, difficulties encountered, self-care, changing attitudes in the daily routine and resuming social interactions. Duration: 30 min
Physical Educator	Importance of creating a physical exercise routine that can performed safely independently. Guidance on exercise intensity based on muscle strength, and instruction on aerobic exercises. The guidelines were based on a booklet with six exercises for muscle strengthening of the lower limbs that could be adapted and individualized depending on the patient’s physical capacity and as a way of progressing in intensity and complexity. Duration: 60 min	The home exercise practice included strengthening exercises 2 to 3 times a week on alternating days, with 2 to 3 sets of 8 to 15 repetitions. Patients were instructed to perform at least 30 min of aerobic exercises daily. Exercises could be distributed in periods of 10 min throughout the day. The exercise prescription was adjusted for each patient based on their functional capacity and feedback, such as changing from standing to silted or lying down, the number of repetitions, load, or technique. Duration: 30 min	
Social Worker	Education on quality of life and basic social needs, community partnerships, and social challenges resulting from pain and disability. Education about social benefits, including free transportation, and health insurance for low-income families. Referrals to Social Service Reference Centres. Guidance on community resources for physical activity, and primary care clinics. Promoting social interactions and expanding social network as a buffer mechanism for pain management. Duration: 30 min	Reflecting on the need for social interaction and avoiding social isolation. Promoting of a healthy lifestyle. The importance of leisure time in parks and public spaces as a distraction from pain. Identifying of social demands and the importance of connection to the social support network as a coping mechanism. Duration: 30 min	

In addition to attending the educational sessions, participants received printed materials containing the educational content from each discipline, exercise instruction, and links to educational videos on the IMREA website.^1^ The use of the content on the IMREA website was strongly emphasized for the home exercise program.

Each educational session began with an interactive discussion about the session objectives, patients’ goals and expectations, feedback on their exercise routine, and general questions. The research team met weekly to review the program logistics and discuss each patient’s needs. Elements of the program, such as exercise prescription and dietary plans, were individualized based on the patient’s feedback, physical limitations, exercise tolerance, and comorbidities. Patients were instructed to complete their exercise prescription for at least 30 min daily, however, no log was required from the participants. The study social workers assisted in identifying local facilities for the daily exercise routine if desired by the patient. We have not monitored analgesic medication use, however, patients were instructed to maintain their prescribed or over-the-counter analgesic regimen.

### Statistical analysis

The baseline characteristics were described as means and standard deviations for continuous variables and percentages for categorical variables. Pain intensity was assessed for each knee (R and L) with the VAS at baseline (Session 1) and at the completion of the program (Session 3). The mean pain level for each knee was tested for normality with the Shapiro–Wilk test. Comparisons before and after the intervention were conducted using the Student’s T-test for dependent samples and the Welch approximation for different variances. For the VAS, a significance of 0.05 was established, and the Cohen’s d effect size was calculated for significant differences. All statistical analyses were conducted with STATA14^®^.

## Results

We recruited 176 participants and 55 were enrolled in the study. The study was conducted from February 2022 through July 2023. All fifty-five subjects completed the Knee-SCHOOL program. Six (10.91%) patients attended one of the sessions online due to the inability to attend the class in person. No adverse events were reported.

Table 2 outlines the characteristics of the participants. The mean age was 67.73 (± 7.73) years; most were females (70.9%), and fifty-one (92.7%) had bilateral KOA. The mean BMI was 32.52 (± 5.99), 65.5% were obese (>30 kg/m^2^), and 96.4% reported an inadequate diet. Most participants had normal bowel function. According to the KOOS, KOA had a more negative impact on sports and recreation and quality of life. Daytime sleepiness, measured by the EES, was uncommon among participants.

**Table 2:** Baseline characteristics of study participants (*n* = 55).

Characteristics	Values
Women, *n* (%)	39, 70.9
Age (years), mean (± SD)	67.73 (7.73)
Education level (n; %)	
Elementary School	8 (14.6)
Middle School	5 (9.1)
High School	19 (34.5)
Technical Degree	1 (1.8)
College Degree	22 (40)
BMI (kg/m^2^), mean (± SD)	32.52 (5.99)
Underweight, *n* (%)	3 (5.5)
Normal, *n* (%)	11 (20.0)
Overweight, *n* (%)	5 (9.1)
Obesity, *n* (%)	36 (65.5)
Pain duration, mean years (± SD)	12.41 (10.17)
Comorbidities, n (%)	
Hypertension	37 (67.3)
Diabetes	20 (36.4)
Hypercholesterolemia	24 (43.6)
Unilateral KOA, *n* (%)	4 (7.3)
Bilateral KOA, *n* (%)	51 (92.7)
Right Knee K & L Classification*, *n* (%)	
0 (absent)	1 (1.8)
I (doubtful)	19 (34.6)
II (mild)	11 (20.0)
III (moderate)	15 (27.3)
IV (severe)	7 (3.6)
Left knee K&L Classification, *n* (%)	
0 (absent)	1 (1.8)
I (doubtful)	21 (38.2)
II (mild)	10 (18.2)
III (moderate)	15 (27.3)
IV (severe)	8 (14.6)
Baseline pain intensity (VAS), mean (± SD)	5.52 (2.1)
Bristol stool scale, *n* (%)	
1	6 (10.9)
2	1 (1.8)
3	35 (63.6)
4	12 (21.8)
5	0
6	1 (1.8)
Body Fat percentage, mean (± SD)	42.17 (9.41)
Skeletal Muscle Index, mean (± SD)	7.79 (1.58)
Low, *n* (%)	6 (10.6)
Normal, *n* (%)	49 (89.1)
Healthy Eating Index, mean (± SD)	78.17 (15.14)
Poor, *n* (%)	15 (27.3)
Needs improvement, *n* (%)	38 (69.1)
Adequate, *n* (%)	2 (3.6)
KOOS, mean (± SD)	
Symptoms	56.95 (19.85)
Activities of daily living	47.70 (22.31)
Pain	44.70 (20.28)
Quality of life	25.68 (19.31)
Sports and recreation	20.18 (20.97)
Epworth sleepiness scale, mean (± SD)	8.49 (5.57)
Unlikely, *n* (%)	32 (61.5)
Possible, *n* (%)	13 (25.0)
Probable, *n* (%)	7 (13.5)

KOA, knee osteoarthritis; n, number; SD, standard deviation; BMI, body mass index; K&L, Kellgren-Lawrence osteoarthritis classification; VAS, visual analogue scale; KOOS, Knee Injury and osteoarthritis outcome score; ADL, activities of daily living; * Two patients had total knee replacement on the right side.

**Figure 1:**
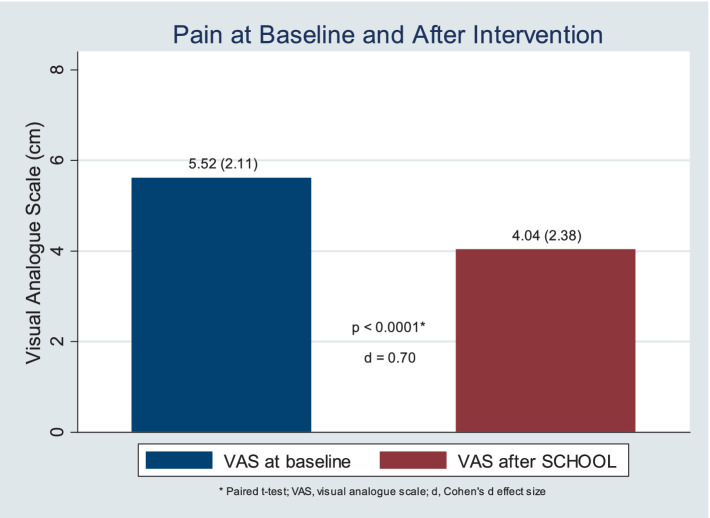
Pain reduction after the Knee-SCHOOL, per visual analogue scale.

The Shapiro–Wilk test demonstrated that the pain intensity at baseline and at completion of the study was parametric (*p* > 0.05) regardless of the side, and the variances were similar along the pain measures. Therefore, the comparisons were conducted with paired Student *T*-tests without corrections. The mean pain level decreased from 5.52 (± 2.11) at baseline (Session 1) to 4.04 (± 2.38) at completion of the study (Session 3), a mean reduction of 1.48 points (± 2.37). This reduction was statistically significant (*p* = 0.0001) with Cohen’s d effect sizes of 0.70 (CI95% 0.32 to 1.07) (Figure 1).

## Discussion

The Knee-SCHOOL is unique because it incorporates a comprehensive face-to-face educational component customized to each patient, and it can be completed in less than 2 weeks. The program covers disease understanding, treatment options, self-management strategies, and coping mechanisms. Additionally, it provides education on physical activity, lifestyle changes, sleep hygiene, nutritional health, and the psychosocial aspects of pain. It includes an exercise plan tailored to the patient’s physical abilities and tolerance. Moreover, it uses booklets and web-based resources to reinforce the educational content covered during the in-person sessions and the home exercise practice (see footnote 1).

Existing self-management programs for KOA often only cover patient education and physical exercise (19–25). The Knee-SCHOOL, however, provides a more comprehensive approach by addressing additional elements, such as nutritional health, sleep hygiene, and psychosocial factors. It is grounded on a solid multidisciplinary team, so all those elements can be adequately addressed.

Physical exercise is a core component in any self-management program for KOA. Exercise programs are safe and effective for pain and strength improvement in KOA (19–25, 38). The Knee-SCHOOL strongly emphasizes the importance of physical activity, and each patient receives an individualized prescription containing stretching and strengthening exercises based on their physical ability. Patients unable to perform exercises in a standing position are taught exercises that can be performed on a chair or lying down. A walking routine is also integrated into the program. The physical therapists and physical educator supervise each patient individually to ensure they learn their exercise routine correctly. In addition, patients are encouraged to access the exercise videos on the IMREA website for their home exercise program.

KOA significantly affects a person’s daily life, causing difficulties in performing activities of daily living (ADL) (39), limited mobility, and reduced participation in work-related activities. Individuals with KOA attribute their difficulty in performing daily activities to symptoms such as pain and limitations in performing essential tasks. In our program, Occupational Therapists assess ADL performance, teach adaptation strategies, and use assistive technology to maintain and promote functionality.

The Knee-SCHOOL prioritizes nutritional health and uses various measures to assess nutritional status. Most participants were found to be overweight or obese, which is consistent with findings in other studies (40). BMI is linked to the severity of pain and should be routinely assessed in pain management educational programs (41). In addition to BMI, our program includes a more in-depth assessment of the patient’s body composition. Some patients were identified as having a low skeletal mass index. We also discovered that a significant number of individuals had unhealthy eating habits, systemic hypertension, and dyslipidemia.

We provide general nutritional education and personalized dietary plans to address these findings. Poor diet is associated with an inflammatory status that can exacerbate symptoms of KOA (42, 43). Proper education on food quality, quantity, meal intervals, and eating habits is also essential to the Knee-SCHOOL (44). Compared to other studies (45, 46), the nutrition education component in our program was much more comprehensive and personalized.

Nursing professionals provided education on the basic physiological functions of the body and other aspects of health education, including an assessment of sleep and education on sleep hygiene. Despite evidence linking sleep duration, restlessness, and sleep quality to KOA (2, 3), sleep is often overlooked in exiting self-management programs for KOA (19, 21, 23–25). We found only two KOA educational programs (45, 47) addressing sleep hygiene, and none included a formal assessment of daytime sleepiness. Restful sleep improves the body’s inflammatory response (48) and can reduce pain and stiffness in KOA, improving quality of life (49). Conversely, lack of sleep or poor-quality sleep can worsen pain perception and affect mood, creating a harmful cycle of insomnia and chronic pain (49). In our study, some participants reported possible or probable excessive daytime sleepiness, and one patient was later diagnosed with sleep apnea. It is essential to routinely assess sleep dysfunction in any self-management program for OA, provide proper education on sleep hygiene, and ensure that patients with more severe symptoms are appropriately evaluated for sleep disorders.

Some educational programs for OA include community resources education (50). Our team social worker provided education on community resources for exercise practice, social benefits, and the importance of having a solid network and social activities, as chronic pain usually causes social isolation and sedentarism.

Another significant component of the Knee-SCHOOL is the education on the psychological aspects of chronic pain. Strong emphasis was given to teaching patients to recognize and reconstruct negative thoughts and behaviors associated with chronic pain, as pain evokes brain modifications (nociplasticity) as an unconscious and involuntary learning process. Such thoughts and behaviors negatively affect patients and their family members (51). Educating and promoting experiences addressing negative ruminations can enhance social support and positive outcomes (52). During the Knee-SCHOOL, patients also received instruction on self-guided relaxation to increase body awareness and relaxation and to improve self-efficacy. Promoting social interactions in the community and within the group was another crucial element, as group-based interventions effectively manage chronic pain (53). The Psychologist promoted group discussions and interactions so participants could share their experiences with the program and reflect on the difficulties and challenges of changing attitudes and behaviors in daily life. Group members supported each other and shared their experiences on solutions to similar challenges.

The multidisciplinary team’s awareness of the importance of lifestyle modifications may play a role in empowering patients to make behavioral changes. Group-based educational intervention allows individuals to develop tools for self-management, enabling them to manage their conditions more effectively (54). Information does not guarantee adherence or modification to different programs (55). What sets the Knee-SCHOOL apart is its focus on lifestyle awareness through a patient-centered multidisciplinary team that includes peer participation from individuals with KOA, facilitating the exchange of information, experiences, and practical strategies. A meta-analysis suggests that strategies aimed at motivating or changing behaviour can enhance adherence to exercise (56), whereas coping counselling and exercise recommendations through audio/video or text message encouragement alone did not significantly improve adherence (56, 57).

All participants received the program well, with no drop-out rates or reported adverse events. Based on the preliminary findings on pain, the Knee-SCHOOL is being considered as the initial step in the treatment plan of patients referred to our institution for KOA-related pain. Also, we observed a reduction in pain level in the short-term, with a Cohen d effect size of 0.7, even though this result should be seen with caution, as no control group was included in this analysis.

Some of the study’s limitations include a small sample size, the absence of a control group, and the lack of measurement of pain beyond the program duration. Additionally, our study does not capture changes in quality of life, functional status, nutritional health, and sleep status following the completion of the program. The Knee-SCHOOL is the initial intervention of a large clinical trial involving other therapies for KOA-related pain. Upon the trial’s completion, data on the long-term effects of the Knee-SCHOOL and the different interventions on pain and additional outcomes will be reported. We have not controlled for analgesic medication use; however, patients reported no changes to the analgesic medication used during the study. Moreover, the Knee-SCHOOL also uses a robust multidisciplinary approach, which may limit its reproducibility in less resourced settings.

## Conclusion

The Knee-SCHOOL employed a comprehensive and team-based educational approach to address pain’s physical, functional, nutritional, and psychological aspects. The program used a patient-centered approach and emphasized collaboration among the healthcare team as a crucial element. It showed promising results in managing KOA-related pain, however future research is also needed to evaluate the long-term impact of Knee-SCHOOL on pain and other outcomes, as well as its applicability in different environments and cultures.

## Data Availability

The data presented in this article is available upon request. Please submit an inquiry to Dr. Marta Imamura on the following email address: marta.imamura@fm.usp.br.

## Footnotes

^1^ https://redelucymontoro.org.br/site/area-do-paciente/materiais-educativos/acesse-aqui-os-videos-da-cartilha-cuidando-do-seu-joelho/
